# Potential Effect of Magnetic Field on *Spodoptera littoralis* (Lepidoptera: Noctuidae): Development, Malform, Reproductivity and DNA Mutagenicity

**DOI:** 10.3390/insects17060637

**Published:** 2026-06-17

**Authors:** Wael Elmenofy, Abd-Elkhalik M. Husseen, Mervat A. Kandil, Hossam S. El-Beltagi, Hosny H. Kesba, Mohamed A. M. Atia

**Affiliations:** 1 Department of Arid Land Agriculture, College of Agricultural and Food Sciences, King Faisal University, Al-Ahsa 31982, Saudi Arabia; hkesba@kfu.edu.sa; 2 Plant Protection Research Institute, Agricultural Research Center, Dokki, Giza 12619, Egypt; esabeegypt@yahoo.com (A.-E.M.H.); dr.mervatkandel@yahoo.com (M.A.K.); 3 Department of Biotechnology, College of Agricultural and Food Sciences, King Faisal University, Al-Ahsa 31982, Saudi Arabia; helbeltagi@kfu.edu.sa; 4 Bioinformatics Laboratory, College of Computing, University Mohammed VI Polytechnic, Ben Guerir 43150, Morocco; mohamed.atia@um6p.ma

**Keywords:** cotton leafworm, hatchability, ISSR, magnetization, molecular markers, polymorphism

## Abstract

*Spodoptera littoralis* (Boisduval, 1833) is one of the most destructive insect pests in Egypt and worldwide. This study was conducted to investigate the impact of exposure to a magnetic field (MF) of 180 milliTesla on the developmental phases of *S. littoralis*, as well as malform, reproductivity, and genomic DNA mutagenicity. The obtained results concluded that the exposure of *S. littorelis* to MF significantly affected the malformation and mortality rates in both larvae and pupae. The MF extended the duration of the pupal stage from approximately 0.8 to 5.9 days compared to the untreated pupae. The adult emergence percentages decreased to 68.0 and 74.0% upon exposure to a magnetic field for 60 and 40 min, respectively. The female fecundity decreased by increasing the exposure duration, yielding (7–10), (6–10), and (3–8) mass eggs per female upon exposure intervals of 20, 40, and 60 min, respectively. Meanwhile, the hatchability percentage diminished with prolonged exposure time, recording 77%, 60%, and 53% for MF exposure durations of 20, 40, and 60 min, respectively, compared to 91% hatchability in the control trial. The genetic characterization employing inter-simple sequence repeat (ISSR) markers disclosed genetic mutagenicity, exhibiting a similarity matrix range from 61.6% to 74.1% for larvae, 59.8% to 68.5% for adults, and 36.2% to 49% for pupae, indicating genetic alteration in treated insects. Hence, these findings highlight the implications and prospective application of a magnetic field of 180 milliTesla as a unique approach in integrated *S. littoralis* control frameworks.

## Simple Summary

The cotton leafworm, *Spodoptera littoralis*, is one of the most damaging crop pests worldwide, causing serious losses in wild plant species, including cotton, vegetables, ornamentals, and different field crops. Current control methods rely heavily on chemical pesticides, which can affect human health, harm the environment, and lead to pesticide resistance. Hence, the current study investigates whether exposure to a specific magnetic field could affect the growth, survival, reproduction, and genetic stability of this insect pest. The results showed that exposure to the magnetic field increased death rates and physical deformities in the insect’s immature stages, delayed development, reduced the number of adults that successfully emerged, and shortened adult lifespan. This study also found evidence of changes in the insect’s genetic material after exposure, suggesting that magnetic fields can influence biological processes at the molecular level. Stronger effects were observed with longer exposure times that ranged from 20 to 60 min. These findings indicate that magnetic fields may provide a promising non-chemical approach for reducing populations of this destructive pest. Such methods could contribute to more sustainable crop protection strategies, helping to reduce dependence on conventional pesticides while supporting environmentally friendly pest management practices.

## 1. Introduction

*Spodoptera littoralis* (Boisduval, 1833) (Lepidoptera: Noctuidae) is a significant economic pest that inflicts damage on numerous agricultural products and is extensively distributed throughout Egypt, the Mediterranean region, Africa, and parts of the Middle East. This highly polyphagous insect pest attacks more than 100 cultivated and wild plant species, including cotton, vegetables, ornamentals, and field crops, causing substantial economic losses through direct feeding damage and reduced crop quality. It results in yield losses ranging from 35% to 55% during the cotton and vegetative stages of the crops [1]. Relying on chemical insecticides for controlling *Spodoptera littoralis* has resulted in several drawbacks, including environmental contamination, insecticide resistance, negative consequences for non-target organisms, and health risks to humans. As a result, creating sustainable and ecologically friendly alternative control methods has emerged as a top research goal [1]. Magnetic fields (MFs) are becoming more widely acknowledged as significant environmental factors that can affect a diverse array of biological processes in living organisms [2,3]. In insects, exposure to magnetic fields has been associated with alterations in development, orientation, behavior, reproduction, longevity, and physiological performance [3]. Different studies have reported that exposure of insects to a magnetic field may affect larval growth, embryonic development, fertility, fecundity, and survival rates in different insect species [3,4,5]. These biological effects have been attributed to several mechanisms, including disruption of cellular signaling pathways, changes in membrane permeability, alteration of ion transport, induction of oxidative stress, and interference with free-radical reactions via the radical-pair mechanism [4]. The magnetic field (MF), a considerable one among many important environmental factors, has a significant influence on living organisms and is capable of affecting a number of biological systems [2,4]. Magnetic fields have been shown to influence the reproductive performance and developmental parameters of *Ephestia kuehniella*, *Pectinophora gossypiella*, and *Drosophila melanogaster*. The influence of magnetic fields on multiple aspects of the biology of the adult Mediterranean flour moth *Ephestia kuehniella* was thoroughly examined [3]. In the same context, the Magnetic Ferrosolution (MFS) was shown to affect some biological aspects of *Pectinophora gossypiella* and reduce the fecundity and fertility of the adult stage [4]. Additionally, Ramirez et al. [5] discovered that *Drosophila melanogaster* subjected to (MF 1 mT) exhibited oviposition reduction and an elevated immature death rate. Also, larval growth was slower in a high magnetic field compared to a geomagnetic field. However, information about the effects of magnetic field exposure on developmental biology, reproductive capacity, and genetic stability of *S. littoralis*, despite its economic importance, is still limited. Moreover, most of the available studies have mainly focused on biological responses [6], while the potential genetic consequences of magnetic field exposure have been relatively less investigated.

Molecular markers have been considered a valuable technique for detecting and evaluating the degree of genetic variability, genetic fidelity, molecular phylogenetics [7], and disease resistance [8].

In insect populations, variations in the techniques of molecular markers have been identified [9]. Within these techniques, microsatellites such as ISSR (inter-simple sequence repeats) have been shown to be effective in detecting genetic variations and differentiating closely related individuals [10]. The ISSR markers have various advantages, such as high reproducibility, extensive coverage of the genome, technical simplicity, and the ability to detect polymorphic DNA regions without prior genomic information [11]. The ISSR markers are characterized by a high variability level, and this has been indicated as a common feature between Lepidoptera genomes, which is used as a fingerprint technique within groups of different organisms [11]. The ISSR is a simple and reliable molecular marker technique that was developed by Zietkiewicz et al. [12]. It has a high polymorphism level and requires no prior investment in primer design. It has been used to amplify multiple loci for the detection of genetic variation within the order Lepidoptera [13]. Consequently, ISSR analysis is a useful method for evaluating potential genetic modifications associated with magnetic field exposure. Although the use of magnetic fields has been proposed as an environmentally safe physical agent with potential applications in integrated pest management programs, their biological and molecular effects on different developmental stages of *S. littoralis* are not sufficiently understood. Especially, little is known about stage-specific susceptibility, developmental abnormalities, reproductive impairment, and possible genomic alterations induced by magnetic field exposure. Therefore, the present study aimed to investigate the effects of a static magnetic field of 180 mT on the biological and genetic properties of *S. littoralis*. The effects of exposure to magnetic fields on the development of larvae and pupae, mortality, malformation, emergence of adults, fecundity, fertility and longevity were studied. The possible genetic changes and DNA polymorphism associated with magnetic field treatment were also evaluated using ISSR molecular markers. The findings of this study provide a better understanding of the biological responses triggered by magnetic fields, as well as information on possible applications of magnetic fields as an ecofriendly part of integrated pest management programs.

## 2. Materials and Methods

### 2.1. Insects

The *S. littorelis* moths (laboratory strain of adult), freshly emerged, were nourished with cotton wool soaked in a 10% sugar solution, which was replaced every 48 h under controlled conditions of 26 ± 1 °C and 75 ± 5% relative humidity at the Bollworms Research Department, Plant Protection Research Institute, Agricultural Research Center. Egg masses were collected and separately confined in sterilized jars (Thermo Fisher Scientific Inc., Waltham, MA, USA), that were covered with muslin covers. Upon larval hatching, fresh and clean castor-bean leaves were provided as a food for larval rearing, pupation, and subsequently, adult emergence. Rearing was performed for three successive generations to ensure pure race specimens. Rearing for the biological investigations aimed to measure and calculate parameters, such as different stages duration, dead ratio, malformation, and adult emergence %, fecundity, and fertility of the resulting females.

### 2.2. Magnetic Power Treatment

Six groups of third instar larvae were prepared for each experiment. Each group consisted of 45 individuals. The first three groups were treated by magnetic power to examine the effects of three times exposures (20, 40, and 60 min) on specific biological aspects, while the second three groups were treated by magnetic power for genetic analysis. The magnetic flux was measured using a magnetizing battery device that generated a magnetic field strength of 180 milliTesla (mT), as specified according to Ahmed et al. [14].

The control group was subjected to the same handling and apparatus conditions as the treated groups, including placement inside the glass tubes and magnetic device, but without activation of the magnetic field (sham exposure).

A magnetic field was generated between two opposing magnetic poles, and the insect-containing glass tubes (15 cm length × 1.5 cm diameter) were positioned centrally within the exposure zone. Magnetic flux density was verified using a calibrated magnetic field meter (±1 mT accuracy) at the center of the exposure region, where the field strength was maintained at 180 mT. Although the glass tubes measured 15 cm in length, the effective exposure area corresponded to the central region between the magnetic poles, where field variation was minimal.

### 2.3. Laval Stage Exposure

Individuals of the first group were carefully housed in a glass tube (15 cm length × 1.5 cm diameter), then the tube was maintained between the 2 poles of a magnetic apparatus with a magnetic field power (180 milliTesla) for 20 min. The second group of larvae was similarly exposed to the same magnetic field power (180 milliTesla) for 40 min, while the third group of larvae was exposed to the same magnetic field power (180 milliTesla) for 60 min. The three remaining groups were reared without any treatment as a control. By the end of treatment, three of the treated and untreated groups were separated and subjected to genetic analysis and investigations using inter-simple sequence repeats (ISSR) polymorphism.

### 2.4. Pupal Stage Exposure

One-day-old pupal-stage *S. littorelis* (120 individuals) were divided into four groups (in tubes 1.5 × 15 cm). Each group contained 30 pupae, and the three groups were exposed to a magnetic field (180 milliTesla power) for three time intervals for 20, 40, and 60 min, as mentioned previously. The fourth group served as a control without any treatment. The pupae were examined every day until the emergence of adults. The duration and mortality rates were ascertained. All newly emerging moths from both the treatment and control groups were sexed.

### 2.5. Adults Stage Exposure

The newly emerging moths of *S. littorelis* (one day old) originated from the laboratory strain. Forty-five pairs of adults were allocated into three groups, each including 15 pairs. Each group was then subdivided into three subgroups, each containing 5 pairs, with 3 replicates for each subgroup (5 females × 5 males). Each group was placed in a glass tube (1.5 cm × 15 cm) for exposure to a uniform magnetic field of 180 milliTesla for three time intervals of 20, 40, and 60 min. A fourth set of moths served as the control group. All cages were provided with only the original diet containing 20% sucrose. Daily examinations of each cage were conducted, and the cumulative egg production per female was determined from the daily tallies of laid eggs. The hatchability and fecundity percentages of the eggs were determined according to Crystal et al. [15] using the following equations:

Hatchability (%) = (No. hatched eggs/No. deposited eggs) × 100

Fecundity (%) = (No. eggs for treated female/No. eggs for untreated female) × 100

### 2.6. Statistical Analysis

All quantitative data were presented as mean ± standard deviation (SD), derived from three replicates per treatment. A one-way analysis of variance (ANOVA) was employed to determine statistically significant differences among magnetic field exposure groups (20, 40, and 60 min) and the untreated control, in accordance with established methodologies [16]. Prior to conducting ANOVA, percentage/proportional variables (e.g., larval and pupal mortality, malformation, adult emergence, fecundity, and hatchability) were subjected to an arcsine square-root transformation to stabilize variances and better approximate normality [17]. The transformed data were subsequently tested for normality of residuals and homogeneity of variances, both of which met the assumptions required for parametric testing. Non-percentage traits (e.g., developmental durations and longevity) were analyzed without transformation. When significant effects were observed (*p* < 0.05), post hoc multiple comparisons were conducted using Tukey’s Honest Significant Difference (HSD) test to identify differences between group means [18]. Additionally, ordinary least squares (OLS) regression was performed to evaluate the explanatory power of exposure time on each biological parameter. For each trait, such as larval mortality, malformation, pupal emergence, fecundity, and hatchability, the regression model’s coefficient of determination (R^2^), F-statistic, and associated *p*-value were reported. All statistical analyses and visualizations were executed using Python (Python Software Foundation. (2024). Python (Version 3.13), https://www.python.org), utilizing the stats models library for ANOVA and regression analysis [19], as well as scipy for statistical computations and seaborn for graphical presentation [20].

### 2.7. Molecular Analysis

#### 2.7.1. DNA Extraction

DNA was extracted from both treated and untreated third instar larvae, pupae, and adults of *S. littorelis* utilizing the G-spin total DNA extraction kit in accordance with the manufacturer’s instructions (INtRON Biotechnology, Seongnam-si, South Korea). Each sample’s DNA concentration was quantified and subsequently standardized to 10 ng/µL in preparation for additional molecular investigations.

#### 2.7.2. Inter Simple Sequence Repeats (ISSR) Polymorphism Analysis

The ISSR-PCR amplification was performed following the methodology described by Luque et al. [13]. Across all treatments, a total of 15 ISSR primers were utilized. The following components were used for each PCR reaction: 25 ng genomic DNA, 1× PCR buffer, 1.5 mM MgCl_2_, 1 μM of each primer, 0.25 mM of each dNTP, and 1 U Go-Taq Flexi polymerase (Promega, Madison, WI, USA).

The PCR program was performed using a GeneAmp PCR System 9700 (Applied Biosystems, Inc., Foster City, CA, USA). PCR was initiated for 5 min at 94 °C, followed by 35 cycles consisting of 94 °C for 1 min, 50 °C for 1 min, and 72 °C for 90 s, with a final extension at 72 °C for 7 min. The generated PCR amplicons were subjected to electrophoresis on a 1.5% agarose gel and imaged utilizing a Gel Doc^TM^ XR+ System (Bio-Rad, Hercules, CA, USA).

#### 2.7.3. ISSR Data Analysis

ISSR bands across all primers were scored visually in binary form (1 = present, 0 = absent) to build a presence/absence matrix for each life stage. Pairwise similarity between samples was computed using Jaccard’s coefficient, J = a/(a + b + c), where “a” is the number of bands shared by both samples, “b” is the number of bands present only in sample 1, and “c” is the number of bands present only in sample 2. Joint absences (0/0) were excluded, as it is standard for dominant markers. The resulting similarity matrices were used to run principal component analysis (PCA). All computations were performed in PAST software (v4).

## 3. Results

### 3.1. Effects of MF on Larvae Duration and Developed Pre-Pupa and Pupa Stages

As shown in Table 1, a magnetic field (MF) at 180 milliTesla power caused malformed, mortality, and prolonged development in the different stages of *S. littorelis* after magnetization of third instar larvae at three time intervals of 20, 40, and 60 min. This caused a significant prolongation of the average duration of the larval stage to 15.9, 16.8, and 18.1 days/larvae compared with 14.6 days in untreated larvae. In the pre-pupae stage, this increased to 2.4, 2.6, and 3.3 days/pre-pupae compared to 2.3 days in the case of untreated individuals. On the other hand, the pupal stage resulting from larvae treated lasted 8.0, 10.3, and 11.1 days/pupae, compared to 7.6 days in the untreated check upon exposure to a magnetic field (MF) at 180 milliTesla power at 20, 40, and 60 min, respectively.

### 3.2. Effects of MF on Larvae and Pupae Mortality and Malformation

The results showed that exposure of *S. littorelis* larvae (third instar larvae) to a magnetic field (MF) with an intensity of 180 mlt power caused noticeable differences in malformed and mortality percentages of larvae and pupae. The effect was clearly observed for *S. ittorelis*, which recorded 11.0, 13.0, and 21.0% mortality and 9.0, 11.0, and 18.0% malformed larvae when exposed for 20, 40, and 60 min, respectively, compared with 4% and 3% mortality and malformed larvae, respectively, for untreated larvae (Table 1). In addition, the pupae resulting from magnetization of third instar larvae were highly affected by mortality and malformation, with 4.0, 7.0, and 9.0% mortality compared to 2.0% in the control, and 7.0, 12.0, and 16.0% pupae malformed compared to 4.0% in the control, when exposed at different time intervals of 20, 40, and 60 min, respectively (Table 1).

### 3.3. Effects of Different Tested Time Magnetizations on Pupae Stage of S. littorelis

#### 3.3.1. Effects of Magnetization on Pupal Duration

The data presented in Table 2 show the effect of magnetic power (MP) on the pupal duration of *S. littorelis*. The pupal stage was significantly elongated to 9.4 days/pupa when exposed to a 20 min period of magnetic power, and the duration increased with increasing exposure time, being recorded as 11.3 days/pupa at an exposure time of 40 min and 14.5 days/pupa at an exposure time of 60 min, compared with 8.6 days/pupa in untreated pupae of *S. littorelis*. The evaluated MF markedly extended the duration needed for the developmental stage (duration of the pupal stage), approximately from 0.8 to 5.9 days, compared to that of those untreated (Table 2). In addition, the pupal mortality percentages were affected by exposure to magnetic power for different times. The effect was observed as 17.0, 26.0, and 32.0% upon exposure to high magnetic power (180 milliTesla) for 20, 40, and 60 min, respectively, compared with 4.0% for untreated pupae (Table 2).

#### 3.3.2. Adult Emergence and Malformed

As shown in Table 2, the adult emergence percentages decreased to 68.0 and 74.0% upon exposure to a magnetic field (MF) at 180 milliTesla power for 60 and 40 min, respectively. However, this percentage increased to 83.0% when the exposure time decreased to 20 min, compared to the untreated pupa, which showed emergence percentages of 96.0%.

On the other hand, a high percentage of malformed adults emerged that resulted from pupae exposed to long durations of 60 min, followed by 40 min; this was estimated to be 11% and 8%, respectively, compared to 3% in the control, and low malformed (2%) was observed as a consequence of exposure to a magnetic field for 20 min (Table 2).

#### 3.3.3. Sex Ratio

The sex ratio was determined using the number of females divided by the total during pupae magnetization. In the control group (untreated), the sex ratio was estimated at 0.51. This ratio increased to 0.63 upon exposure of the *S. littorelis* pupal stage with a long duration time (60 min) of 180 mlt magnet power, while it relatively decreased to 0.49 upon exposure of pupae to a magnetic field for 40 min. However, no effect appeared between the control and pupae that were magnetized for 20 min, as shown in Table 2, as nearly similar values (0.51 and 0.52) were recorded for untreated and 20 min treated pupae, respectively.

### 3.4. Effects of Magnetization on Adult Stage of S. littoralis

#### 3.4.1. Ovipositional Periods

The pre-oviposition period for female *S. littorelis* was 2.9 and 3.1 days when subjected to magnetic fields twice, at 20 and 40 min, respectively, while it increased to 4.9 days when exposed for 60 min, compared with 2.6 days in non-treated adults (Table 3). The oviposition period showed a gradual significant reduction to 9.0, 6.6, and 4.0 days when exposed to magnetic fields for 20, 40, and 60 min, respectively, compared with 9.3 days in the control.

#### 3.4.2. Adult Longevity

Table 3 clearly demonstrates that the lifespan reduction of females exposed to a magnetic field for 40 and 60 min was 12.7 and 10.2 days for females compared to 14.3 days in the control group. No significant difference was observed between *S. littoralis* adults’ lifespan upon exposure to a magnetic field at 180 milliTesla power for 20 min, which recorded 14.5 days compared to the control (14.3 days).

#### 3.4.3. Potential Reproductivity

Table 3 illustrates that highly significant differences were identified between reproductive adults subjected to varying durations of magnetization and the control group. The average fecundity of females subjected to three exposures was (7–10), (6–10), and (3–8) mass eggs per female when the adult was exposed to 180 milliTesla magnetic fields for 20, 40, and 60 min, respectively, compared to (8–13) mass eggs/female in the case of the control experiment. Meanwhile, the percentage of hatchability decreased by increasing the exposure time, recorded at 77, 60, and 53%, when the adult was exposed to a 180 milliTesla magnetic field for 20, 40, and 60 min, respectively, compared with 91% hatchability in the control experiment.

#### 3.4.4. ISSR Analysis

The effect of magnetic power exposure periods on the DNA mutagenesis of *S. littorelis* adult, larvae, and pupae was determined using the ISSR marker system. A total of 12 ISSR primers have been used in the amplification of all the treatments of larvae, adults, and pupae. The 12 ISSR primers produced a total of 168 scorable bands, with an average of 14 bands for each primer in larvae; 178 scorable bands, with an average of 14.8 bands/primer in adult; and 223 scorable bands, with an average of 18.6 bands/primer in pupa. The data are presented in Table 4. The number of amplified DNA fragments per primer in every group of samples ranged from 7 bands (primer ISSR 16) to 22 bands (primer ISSR 14) in larvae, 5 bands (primer ISSR 6) to 21 bands (primer ISSR 3) in adults, and 11 bands (primer ISSR 18) to 29 bands (primer ISSR 19) in pupae. Furthermore, the number of polymorphic bands per primer in every group of samples ranged from 1 band (primer ISSR 15 and ISSR 16) to 16 bands (primer ISSR 18) in larvae, 1 band (primer ISSR 6) to 15 bands (primer ISSR 3) in adults, and 0 bands (primer ISSR 14) to 8 bands (primer ISSR 10 and ISSR 18) in pupae.

#### 3.4.5. Analysis of Molecular Phylogeny

**Figure 1:**
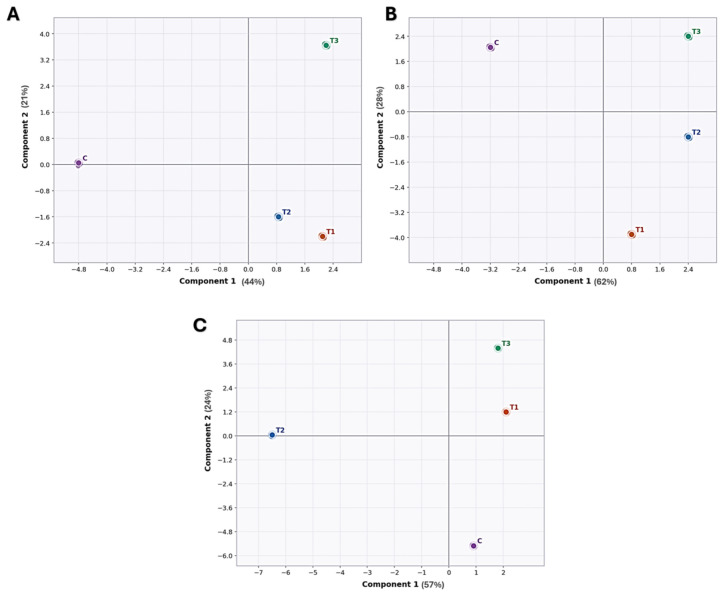
Principal component analysis (PCA) of ISSR profiles of *Spodoptera littoralis* exposed to a magnetic field of 180 mT for 20, 40, and 60 min compared with the untreated control. (**A**) Larvae, (**B**) adults, and (**C**) pupae. C: control; T1: 20 min exposure; T2: 40 min exposure; T3: 60 min exposure. Each panel shows the two-dimensional projection of the ISSR-derived binary matrix on the first two principal components, illustrating treatment-associated divergence from the control in each life stage.

A PCA graph was plotted for the control and three treatments of larvae, adults, and pupae of *S. littorelis* based on the UPGMA cluster analysis of ISSR data. In larvae, the PCA plot separated both the control and treatment 3 alone, while plots for treatment 1 and treatment 2 closed. On the other hand, the PCA of adults grouped the control sample and the first treatment together, while treatments 2 and 3 were plotted closely to each other. The PCA of the pupae plotted treatment 3 separately, whereas treatment 2 was positioned slightly farther from treatment 1 and the control sample. PCA revealed clear separation between the control (C) and treatment groups (T1, T2, and T3) across all three life stages, primarily driven by Component 1, which explained 44%, 62%, and 57% of the variance in larvae, adults, and pupae, respectively. Meanwhile, component 2 accounted for a smaller but complementary proportion of the variance (21%, 28%, and 24%), collectively highlighting consistent treatment-induced shifts regardless of life stage (Figure 1). Similarity indices based on Jaccard’s coefficient were generated from the ISSR analysis data of larvae, adults, and pupae (Table 5). The values of the similarity matrix range from 61.6% to 74.1%, 59.8% to 68.5%, and 36.2% to 49%, for larvae, adults, and pupae, respectively.

## 4. Discussion

Many types of radiation, like gamma rays, X-rays, and magnetic fields, have been shown to have a negative impact on insects and many different organisms. A wide range of insects have been reported to be affected by magnetic fields in terms of oviposition, development, orientation, behavior, and fecundity [21]. Moreover, exposure to a static magnetic field inflicted damage on larval cellular DNA, and somatic cells deficient in standard DNA repair mechanisms were unable to complete cell division, leading to the embryonic mortality of mutant larvae [21]. Hence, from a pest management point of view, the relationship between the developmental responses of *S. littorelis* and exposure time to high magnetic fields is crucial to understanding the biology and life histories of insects. The results obtained in the current research recorded a relationship between an increase in exposure time, 20, 40, and up to 60 min of *S. littorelis*, to a high magnetic field (180 milliTesla power) on different insect life stages (larvae, pupae, and adults). Upon exposure to a 180 milliTesla magnetic field, elongation in larval development was detected. Also, a latent effect on the longevity time of the adult stage of *S. littorelis* resulted from either pupae treatment or adults being directly exposed to a magnetic field (magnetization). This effect was also detected and showed a gradual decrease with increasing exposure durations of 20, 40, and 60 min.

The increase in exposure time to magnetic fields showed an apparent effect on mortality and malformation in larval, pupal, and adult stages. Also, the effect was apparent in the reduction of the number of eggs laid by magnetized females (fecundity) and egg hatching (fertility). Hatching was highly decreased with increasing magnetic field exposure times of 40 and 60 min. These results agree with Barry et al. [6], as they recorded that larval rearing development exposed to a strong magnetic field was slower than that observed without treatment.

In addition, the fertility of the Mediterranean flour moth, *Ephestia kuehniella*, significantly diminished when subjected to a magnetic field [3]. Moreover, Said et al. [22] demonstrated that magnetic flux significantly influenced the fecundity and reduced the hatchability of eggs in the pink bollworm, *Pectinophora gossypiella*. In the same context, Kandil et al. [4] documented that the Magnetic Ferrosolution significantly decreased the fecundity and fertility of *P. gossepeilla*. Moreover, the duration of exposure to static magnetic fields (SMFs) adversely affected the fecundity and fertility of *Sitobion avenae* adults treated to 0.176 T for 30 min and 0.065 T for 60 min [23]. It was reported that exposing *E. kuehniella* adults to escalating concentrations of MFs dramatically decreased daily egg production and progeny yield [3].

Previously, an increased mutation rate in a population of Drosophila exposed to a magnetic field 10–12 times higher than a geomagnetic field had been used to evaluate the static magnetic field effect on mutagenicity [24].

Different molecular markers have been successfully used to detect genetic mutations upon exposure to environmental stress. In a study by Sayed et al. [25], six RAPD markers were used to evaluate the variation caused by sterilizing and substerilizing doses of gamma-irradiation on sterility in male parents and F1 *Ceratitiis capitata* pupae. The results of RAPD-PCR profiles revealed a variability between normal males and irradiated *Ceratitis capitata* pupae and their F1. Similar findings were recorded by Zahran et al. [26], who used four RAPD markers to estimate the variation caused by sterilizing and substerilizing doses of gamma-irradiation on sterility in full-grown male pupae of the peach fruit fly *Bactrocera zonata,* and the result of RAPD-PCR amplification profiles revealed a variability between male pupae of *Bactrocera zonata*. The ISSR molecular marker was also used extensively as an effective tool to differentiate between treated and untreated insect populations. Hence, in the current study, by using 12 ISSR primers, the number of amplified DNA fragments per primer in every group of samples ranged from 7 to 22 bands in larvae, 5 to 21 bands in adults, and 11 to 29 bands in pupae. These amplicons confirmed the suitability of the selected ISSR primers to differentiate between treated and non-treated insects, which reflects the mutagenicity represented upon MF treatments. These results were in agreement with a recent study that evaluated the impact of short-term magnetic field exposure on duration and genetic changes in *Agrotis ipsilon* larvae [14]. Upon using ISSR markers, a mutagenic change was observed, with a polymorphism rate of 74.13%. Furthermore, ISSR markers were successfully used to evaluate the effect of the sublethal and lethal concentrations of nano-insecticides on oxidative stress enzyme activity, reproductive activity, and genetic mutagenicity [27]. The markers revealed polymorphic differences in ISSR patterns due to DNA damage or changes in DNA caused by exposure to insecticides. In conclusion, the present study offers significant insights into the biological and genetic impacts of magnetic fields on *S. littoralis* across various life stages. The results indicated that the exposure of *S. littorelis* larvae to a magnetic field (MF) at 180 mlt power significantly affected the rates of malformation and mortality in both larvae and pupae. Moreover, exposure to MFs adversely affected female fecundity, the mass of eggs laid per female, and the hatchability percentage. Moreover, the ISSR markers effectively detected genetic mutagenicity and polymorphism caused by magnetic exposure with extended exposure durations (40 and 60 min).

Markedly, the pupal ISSR profiles showed lower similarity compared to the controls (36.2–49.0%) than larvae or adults, which indicates heightened stage-specific sensitivity. These unexpected results are likely caused by intense metamorphic remodeling with heightened DNA replication and repair demands, which consequently increase susceptibility to MF-induced damage. On the other hand, pupae reduced their antioxidant capacity and failed to activate any behavioral/nutritional stress mitigation. Further contributions include incremental damage from prior larval exposure and strong physiological influences (longer duration, higher mortality, etc.). Nevertheless, the SSR-PCR system is dominant; our findings require further mechanistic verification to prove any induced genomic alterations.

Reportedly, both static and low-frequency magnetic fields disturb the kinetics of electron transport and free-radical recombination via a mechanism called the “radical pair” [21]. This mechanism leads to an accumulation of reactive oxygen species (ROS), whose rise in insect tissues is a key inducer of oxidative DNA lesions and disturbance of DNA-repair fidelity, altogether altering primer-binding sites and amplification outcomes in ISSR-PCR [3]. Therefore, this indirect pathway is one of the most logical explanations for the amplicon’s gain/loss pattern observed in our varied samples (larvae, pupae, and adults), and it agrees with the other concurrent biological influences reported here, such as malformation, developmental delay, and reduced hatchability. All these observed effects are classical downstream consequences of oxidative damage in lepidopteran insects [25,27].

On the other hand, and in the context of the disruption of cellular and metabolic processes as an indirect mechanism, MF exposure is reported to alter membrane permeability, Ca^2+^ homeostasis, and the activity of antioxidant enzymes in insects [3,4]. These indirectly compromise genomic stability by impairing DNA repair machinery, hindering cell-cycle checkpoints, and amplifying ROS-mediated lesions during DNA replication, particularly during the metabolically active developmental stage transitions.

These results underscore the significance of magnetic fields as a potent environmental stressor capable of inducing genetic alterations in insects, offering an innovative strategy for pest management. Future research should investigate the long-term impacts of magnetic field exposure on insect populations, specifically on reproductive success, resistance development, and ecological ramifications. Hence, this stressor may be used as an effective tool for *S. littoralis* control through an integrated pest management program. Furthermore, broadening studies to examine the possible mutagenicity effect of magnetic fields in additional pest species would provide significant insights into the wider use of this methodology as a safe and sustainable strategy in insect pest management programs.

Ultimately, our ISSR data should therefore be interpreted as treatment-associated genomic changes (fingerprint) rather than direct evidence of a specific mutagenic mechanism. Our future work will focus on combining ISSR fingerprinting with the comet assay and antioxidant enzymes quantifications to formally discriminate between the direct DNA-targeting effects and indirect ROS-mediated effects under MF treatment of the *S. littoralis*.

## 5. Conclusions

In conclusion, the present study offers significant insights into the biological and genetic impacts of magnetic fields on *S. littoralis* across various life stages. The results indicated that the exposure of *S. littorelis* larvae to a magnetic field (MF) at 180 mlt power significantly affected the rates of malformation and mortality in both larvae and pupae. Moreover, exposure to MF adversely affected female fecundity, the mass of eggs laid per female, and the hatchability percentage. Moreover, ISSR markers effectively detected genetic mutagenicity and polymorphism caused by magnetic exposure with extended exposure durations (40 and 60 min). Future studies should focus on evaluating the long-term effects of magnetic field exposure on insect populations, particularly regarding reproductive performance, the potential development of resistance, and broader ecological consequences. These findings suggest that magnetic field exposure could serve as a promising component of integrated pest management (IPM) programs for the control of *Spodoptera littoralis*.

## Figures and Tables

**Table 1:** Effect of magnetic field times on larval stage of *S. littoralis*.

Time of Magnetic Exposure (min.)	Effect of Magnetic Field on Larval Stage					Pupal Stage			
	Duration (Mean ± SD)	From Exposure to Pre-Pupae		Duration of Pre-Pupae	% Pupal Formed	Duration (Mean ± SD)	Dead %	Malformed %	Reduction % In Larval and Pupal Stages
		Dead %	% Malformed						
20	15.9 ± 2.60 ^c^	11.0 ± 8.08 ^c^	9.0 ± 7.39 ^b^	2.4 ± 0.35 ^a^	80.0 ± 10.33 ^b^	8.0 ± 0.87 ^c^	4 ± 6.20 ^c^	7.0 ± 8.5 ^b^	31.0 ± 11.94 ^c^
40	16.8 ± 0.43 ^b^	13.0 ± 8.68 ^b^	11.0 ± 7.10 ^b^	2.6 ± 0.52 ^a^	76.0 ± 11.52 ^c^	10.3 ± 1.21 ^b^	7 ± 8.07 ^b^	12.0 ± 10.28 ^a^	43.0 ± 12.78 ^b^
60	18.1 ± 2.25 ^a^	21.0 ± 10.52 ^a^	18.0 ± 9.92 ^a^	3.3 ± 0.17 ^a^	61.0 ± 12.59 ^d^	11.1 ± 0.87 ^a^	9 ± 7.2 ^a^	16.0 ± 11.59 ^a^	64.0 ± 12.39 ^a^
Control	14.6 ± 12.46 ^d^	4.0 ± 5.06 ^d^	3.0 ± 4.40 ^c^	2.3 ± 0.17 ^a^	93.0 ± 6.59 ^a^	7.6 ± 0.52 ^d^	2 ± 4.43 ^d^	4.0 ± 6.20 ^b^	13.0 ± 8.68 ^d^
	F = 15.23; df = 3; *p* = 1.14 × 10^−3^	F = 28.45; df = 3; *p* = 1.28 × 10^−4^	F = 11.36; df = 3; *p* = 2.96 × 10^−3^	F= 2.18; df = 3; *p* = 1.68 × 10^−1^	F = 33.67; df = 3; *p* = 6.93 × 10^−5^	F = 12.98; df = 3; *p* = 3.93 × 10^−3^	F = 19.77; df = 3; *p* = 4.67 × 10^−4^	F = 28.44; df = 3; *p* = 1.28 × 10^−4^	F = 40.12; df = 3; *p* = 3.62 × 10^−5^

Values are presented as mean ± standard deviation (SD), n = 3. Different lowercase letters within the column indicate statistically significant differences among treatments according to Tukey’s HSD test at *p* < 0.05.

**Table 2:** Effect of magnetic field times on pupal stage of *S. littoralis*.

Time of Magnetic Exposure (min.)	Effect of Magnetic Exposure on Pupal Stage					
	Duration in Day	Increased in Duration (Days)	Dead %	% Adult Emergence	Malformed Adult %	Sex Ratio as Female
20	9.4 ± 0.35 ^a^	0.8	17.0 ± 11.87 ^b^	83.0 ± 11.88 ^ab^	2.0 ± 4.43 ^d^	0.52 ± 0.03 ^a^
40	11.3 ± 0.43 ^a^	2.7	26.0 ±13.40 ^a^	74.0 ± 13.40 ^bc^	8.0 ± 8.58 ^b^	0.49 ± 0.02 ^a^
60	14.5 ± 1.21 ^a^	5.9	32.0 ± 14.75 ^a^	68.0 ± 13.78 ^c^	11.0 ± 9.89 ^a^	0.63 ± 0.04 ^a^
Control	8.6 ± 0.57 ^a^	-	4.0 ± 6.18 ^b^	96.0 ± 6.21 ^a^	3.0 ± 5.39 ^c^	0.51 ± 0.02 ^a^
	F = 2.25; df = 3; *p* = 1.60 × 10^−1^		F = 29.25; df = 3; *p* = 1.16 × 10^−4^	F = 21.56; df = 3; *p* = 3.45 × 10^−4^	F = 132.61; df = 3; *p* = 3.69 × 10^−7^	F = 0.31; df = 3; *p* = 8.18 × 10^−1^

Values are presented as mean ± standard deviation (SD), n = 3. Different lowercase letters within the column indicate statistically significant differences among treatments according to Tukey’s HSD test at *p* < 0.05.

**Table 3:** Effect of magnetic field times on the longevity, reproduction, and fertility for adult stags of *S. littoralis*.

Time of Magnetic Exposure (min.)	Pre- Oviposition	Oviposition	Post- Oviposition	Fecundity and Fertility of *S. littorelis* (Adults)		
				Longevity Female	Range of Masse Eggs Laid	Hatchability %
20	2.9 ± 0.17 ^b^	9.0 ± 0.87 ^a^	2.6 ± 0.35 ^ab^	14.5 ± 2.08 ^a^	7–10 ^a^	77.0 ± 10.92 ^a^
40	3.1 ± 0.35 ^ab^	6.6 ± 0.52 ^a^	3.0 ± 0.17 ^a^	12.7 ± 2.25 ^a^	6–10 ^b^	60.0 ± 12.65 ^a^
60	4.9 ± 0.52 ^a^	4.0 ± 0.35 ^a^	1.3 ± 0.17 ^c^	10.2 ± 1.21 ^a^	3–8 ^c^	53.0 ± 12.90 ^b^
Control	2.6 ± 0.35 ^b^	9.3 ± 1.04 ^a^	2.4 ± 0.36 ^ab^	14.3 ± 2.25 ^a^	8–13 ^a^	91.0 ± 8.07 ^a^
	F = 5.46; df = 3; *p* = 2.45 × 10^−2^	F = 2.81; df = 3; *p* = 1.08 × 10^−1^	F = 7.21; df = 3; *p* = 1.16 × 10^−2^	F = 2.47; df = 3; *p* = 1.36 × 10^−1^	F = 30.08; df = 3; *p* = 1.05 × 10^−4^	F = 13.83; df = 3; *p* = 1.57 × 10^−3^

Values are presented as mean ± standard deviation (SD), n = 3. Different lowercase letters within the column indicate statistically significant differences among treatments according to Tukey’s HSD test at *p* < 0.05.

**Table 4:** Primer names, polymorphism percentages, and total number of bands of ISSR primers for adults, larvae, and pupae of *S. littoralis*.

Primer	Adult		Larvae		Pupa	
	Polymorph. (%)	Total Bands	Polymorph. (%)	Total Bands	Polymorph. (%)	Total Bands
ISSR 3	71.4	21	75.0	12	33.3	12
ISSR 4	66.7	12	69.2	13	36.8	19
ISSR 6	20.0	5	45.5	11	6.3	16
ISSR 8	77.8	9	72.7	11	33.3	18
ISSR 10	28.6	14	71.4	14	40.0	20
ISSR 13	52.9	17	28.6	21	5.9	17
ISSR 14	41.2	17	27.3	22	0.0	23
ISSR 15	11.8	17	8.3	12	12.0	25
ISSR 16	15.0	20	14.3	7	5.2	19
ISSR 18	70.6	17	84.2	19	72.7	11
ISSR 19	31.3	16	25.0	16	3.5	29
ISSR 20	53.8	13	80.0	10	21.4	14
Total	—	178	—	168	—	223
Mean	45.1	14.8	50.1	14.0	22.5	18.6

**Table 5:** Similarity matrix of adults, larvae, and pupae between the control and the three treatments based on Jaccard’s coefficient.

	Adults				Larvae				Pupae			
C	-	-	-	-	-	-	-	-	-	-	-	-
T1	66.0	-	-	-	63.3	-	-	-	49.0	-	-	-
T2	59.8	64.9	-	-	68.0	74.1	-	-	44.7	40.8	-	-
T3	63.2	64.1	68.5	-	61.6	70.0	70.5	-	40.0	40.6	36.2	-

C: the control; T1: exposed to magnetic field power for 20 min; T2: exposed to magnetic field power for 40 min, and T3: exposed to magnetic field power for 60 min.

## Data Availability

All analyzed data are located within the manuscript main text.
